# Quantitative Analysis of Conjunctival and Retinal Vessels in Fabry Disease

**DOI:** 10.1155/2019/4696429

**Published:** 2019-04-10

**Authors:** Andrea Sodi, Chiara Lenzetti, Daniela Bacherini, Lucia Finocchio, Tommaso Verdina, Isabella Borg, Francesca Cipollini, Fatema Ullah Patwary, Ilaria Tanini, Claudia Zoppetti, Stanislao Rizzo, Gianni Virgili

**Affiliations:** ^1^Department of Surgery and Translational Medicine, Eye Clinic, Careggi Teaching Hospital, University of Florence, Florence, Italy; ^2^Institute of Ophthalmology, University of Modena and Reggio Emilia, Modena, Italy; ^3^Department of Pathology, The Medical School, University of Malta, Msida, Malta; ^4^Department of Pathology, Medical Genetics Unit, Mater Dei Hospital, Msida, Malta; ^5^Referral Center for Cardiomyopathies, Careggi Teaching Hospital, Florence, Italy; ^6^Department of Informatic Engineering, University of Siena, Siena, Italy

## Abstract

Fabry Disease (FD) is a rare X-linked lysosomal storage disorder characterized by systemic and ocular involvement. It has been described an increasing in retinal and conjunctival vessel tortuosity and this feature represents an important marker for the disease. Currently, there is not an objective method to measure and quantify this parameter. We tested a new semi-automatic software measuring retinal and conjunctival vessel tortuosity from eye fundus and conjunctival digital images in a group of FD patients. We performed an observational case-control study evaluating three mathematical parameters describing tortuosity (sum of angle metric [SOAM], product of angle distance [PAD], triangular index [I2e]) obtained from fundus and conjunctival pictures of 11 FD patients and 11 age and sex-matched controls. Both eyes were considered. Mann–Whitney test was used to compare the FD group versus the control group and, within the FD group, male versus female patients. Linear regression analysis was performed to evaluate the possible association of retinal and conjunctival vessels tortuosity parameters with age and with specific markers of systemic disease's progression. The tortuosity parameters (SOAM, PAD and I2e) were significantly higher in retinal vessels and in conjunctival nasal vessels in FD patients in comparison with the controls (*p*=0.003, *p*=0.002, *p*=0.001 respectively for retina) (*p*=0.023, *p*=0.014, *p*=0.001 respectively for nasal conjunctiva). No significant association was found between retinal and conjunctival tortuosity parameters and increasing age or systemic involvement markers. Vessel tortuosity represents an important clinical manifestation in FD. A computer-assisted analysis of retinal and conjunctival vasculature demonstrated an increased vessels tortuosity in patients affected by Fabry disease. This non-invasive technique might be useful to help the diagnosis in early stages, to establish disease severity and monitor its progression.

## 1. Introduction

Fabry disease (FD) is an X-linked recessive inborn error of glycosphingolipid metabolism resulting from deficient or absent activity of the lysosomal exoglycohydrolase *α*-galactosidase A (*α*-Gal A) [1]. Affected patients cannot effectively metabolize membrane glycosphingolipids (GSLs), particularly globotriaosylceramide (Gb3), resulting in its progressive accumulation in lysosomes and cytoplasm in a variety of cells, including capillary endothelial cells, renal cells (podocytes, tubular cells, glomerular endothelial cells, mesangial and interstitial cells), cardiac cells (cardiomyocytes and fibroblasts) and nerve cells [2].

The primary disease process starts in infancy, and clinical features of FD usually appear in childhood and adolescence.

The prevalence of FD has been estimated to range between 1 in 40.000 and 1 in 117.000 male births but the true prevalence maybe higher, as highlighted by Italian and Taiwanese new-born screening studies [3]. FD is caused by mutations in the GLA gene, located on the long arm of X-chromosome (Xq22) [4]. As in all X-linked disorders the more severe phenotype usually is manifested in affected males [4]. The clinical manifestations are characterized by cardiac and neurological dysfunctions, cerebrovascular disease, renal insufficiency, hearing impairment and ophthalmic involvement [4–7].

Specific therapy with recombinant human *α*-galactosidase represents an important milestone in the management of Fabry disease [8]. Two forms of recombinant enzyme have been approved in Europe: agalsidase-*α* and agalsidase-*β*, both administered as intravenous infusion biweekly [9]. There is an emerging consensus that Enzyme Replacement Therapy (ERT) has a limited impact on the long-term outcomes, however expert physicians have recommended that ERT should be initiated as early as possible [8, 9]. This observation is in accordance with the hypothesis that glycolipid clearance is most therapeutically effective before secondary, irreversible tissue damage has occurred [9–11]. Alternative therapeutic strategies include chaperon therapy, that offers promises in patients with specific mutations, which result in misfolding and premature degradation of mutant *α*-Gal A [12].

**Figure 1:**
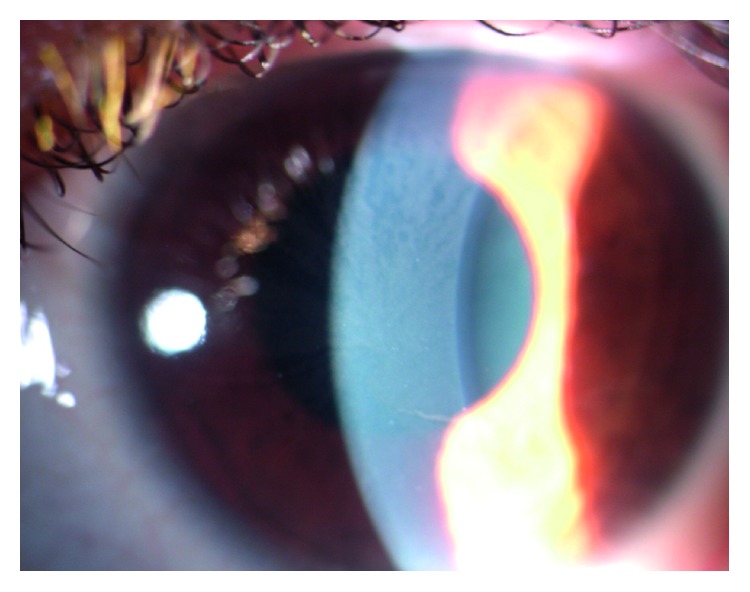
Picture of the anterior segment of a patients included in our study. It is evident a typical form of cornea verticillata.

The ocular signs are important markers in Fabry disease. Cornea verticillata occurs in over 70% of patients and consists in sub-epithelial corneal deposits with a spoke-like appearance. It has been considered the most reliable ophthalmological marker for diagnosing Fabry disease [13] (Figure 1). Lens opacities can usually be observed with two specific manifestations like anterior capsular or subcapsular cataract, generally bilateral and wedge-shaped, and radial posterior subcapsular cataract (Fabry Cataract) [14–21].

Another ocular manifestation of FD is represented by retinal and conjunctival vessel tortuosity, as well as more rarely aneurysms of the conjunctival vessels especially in male patients [15, 22]. Usually ocular abnormalities do not cause significant visual impairment but they may be very useful for early diagnosis, monitoring of disease progression and response to treatment. Unfortunately ocular changes are very difficult to be quantified. Infact, corneal deposits are usually very pale and thin and can be poorly imaged [13]. On the other hand Fabry cataract is relatively rare and the evaluation of vascular abnormalities is very subjective and poorly quantifiable.

Recently the quantitative evaluation of vascular changes in various retinal disorders has been attempted by means of specific software [23–27]. In a previous paper our group measured retinal vessel tortuosity in FD patients by means of dedicated software [28]. To date, conjunctival vessel tortuosity in FD patients has never been quantified. In the present work we used a software developed for retinal vessel tortuosity analysis in order to investigate the course of both retinal and conjunctival vessels in FD, and to try to quantify vessel tortuosity. The purpose of our study is to evaluate the clinical use of a possible quantitative analysis of the retinal and conjunctival vessels in FD as a diagnostic tool and a predictive indicator of systemic abnormalities.

## 2. Materials and Methods

### 2.1. Study Population

Eleven patients (5 males and 6 females) with a clinical diagnosis of FD were included in the study. The mean age was 51.7 (SD: 15.6; range 30–71) years. Ten patients were recruited through Fabry Disease Referring Centre of Careggi Teaching Hospital in Florence, Italy. One female patient was recruited through the Department of Medical Genetics, Mater Dei Hospital, Malta.

The diagnosis of FD was formulated on the basis of the clinical features of the disease, the deficiency of *α*-GAL A in plasma and leucocytes, family history and the presence of GLA gene mutations.

Exclusion criteria were Snellen best corrected visual acuity <0.8, myopia ≥6 diopters, hyperopia ≥3 diopters, astigmatism ≥1.5 diopters, relevant media opacities or retinal disorders. All patients included in our study were diagnosed with FD and did not suffer from any other concurrent significant medical condition. The control group consisted of 11 healthy subjects (5 males and 6 females) recruited from the medical and nursing staff working at the Eye Clinic of the University of Florence, and their relatives (8 and 3 respectively). All joined the study on a voluntary basis. The age range was 30–68 years with a mean age of 46.5 ± 14.5 years. The study was approved by the local Institutional Revision Boards and was in agreement with the principles of the Helsinki Declaration.

All the patients underwent a comprehensive ophthalmological examination, including biomicroscopy of the anterior segment and fundoscopy after dilatation. The presence of conjunctival or retinal vessel tortuosity, cornea verticillata or cataract was subjectively evaluated by specifically trained ophthalmologists (AS, CL) and these ophthalmological findings were carefully imaged.

According to our previous paper [20] some established parameters were considered representative of systemic involvement such as glomerular filtration rate (GFR mL/min), New York Heart Association scale (NHYA), interventricular Maximum Wall Thickness (MWT mm) and neurological manifestations (history of TIA/stroke). The estimated glomerular filtration rate was determined by the 4-variable Modification of Diet in Renal Disease (MDRD) equation, and the interventricular septum diameter was assessed by echocardiography [29].

The main clinical data of our series are summarized in Table 1. In the Table the presence of cornea verticillata and the detected mutations of our patients are also reported.

### 2.2. Vascular Imaging

**Figure 2:**
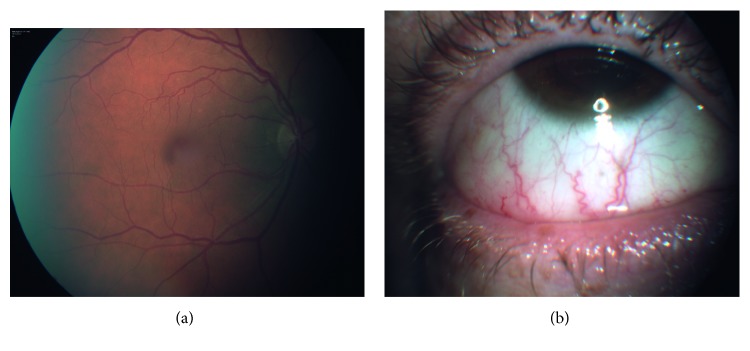
Pictures of retinal and conjunctival vasculature network of a patient included in our study.

We imaged retina and bulbar conjunctiva according to a standardized protocol. First a pharmacological mydriasis was induced using Tropicamide 1% eye drop, and posterior pole pictures were obtained using a fundus camera with a 45° field of view centred on the macula (Retinographs TF 450 Plus, Carl Zeiss, Dublin, CA, USA). For each subject three images were taken of each sector of bulbar conjunctiva (nasal, temporal, superior and inferior sector) of both eyes, by means of Retinograph TF 450 Plus (Carl Zeiss, Dublin, CA, USA). For each sector, the best quality image was selected for processing by three senior investigators (CL, DB, TV). A sample of retinal and conjunctival vascular network of one of our patients is shown in Figure 2. A semi-automated analysis was performed on digital retinographs and conjunctival pictures, using the dedicated software specifically engineered by the Department of Informatic Engineering of the University of Siena [28]. The images processed by the software were TIFF format files which uselossless compression, with a resolution of 1536 × 1024 pixels and colour depth of 8 bits. From every digitalized retinograph and conjunctival picture, three senior investigators (CL, DB, TV) extracted the most tortuous vascular segments (5 for the retina and 3 for each conjuctival sector) using the mouse to select 3 locations along its course. The examiners were blinded about patients' clinical conditions.

### 2.3. Procedure for the Analysis of Retinal and Conjunctival Pictures

The length of the retinal segment chosen for analysis was approximately between 350 and 500 pixels. As for the conjunctival images the length of the segment chosen for analysis was approximately between 250 and 400 pixels. After the selection of the vascular segments of retinal and conjunctival vessels the software automatically calculated three tortuosity parameters: Sum of angles metric (SOAM), Product of angle distance (PAD) and Triangular Index (I2e). Further mathematical details of the chosen parameters have been discussed elsewhere [28].

### 2.4. Statistical Analysis

Mann–Whitney test was used to compare the FD group versus the control group, both for fundus and conjunctival vessels and then males versus females, both in FD and control group. No correction for multiple testing was performed since we always compared two groups. Linear regression analysis was first used to evaluate the possible association between retinal and conjunctival vessel tortuosity indexes, the possible association of retinal vessel tortuosity parameters with increasing age and in FD the possible association of retinal vessel tortuosity indexes with GFR, IVSD and MSSI, in the cases where these data were available.

A probability of *p* < 0.05 was considered statistically significant. All statistical analyses were carried out using the SPSS (Statistical Package for Social Sciences, Chicago, USA) software for Windows (Version 13.0).

## 3. Results

**Figure 3:**
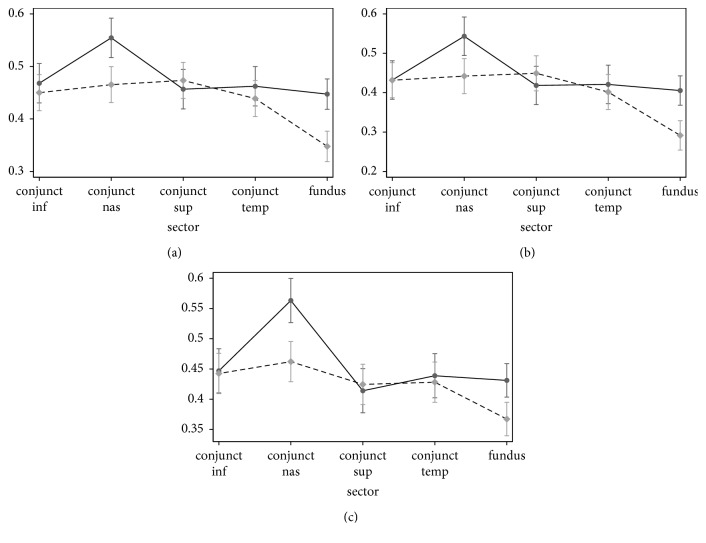
Conjunctival (four different sectors) and retinal fundus vessel to rtuosity parameters in FD and controls. The graphs show a higher tortuosity of the conjunctival vessels in the nasal sector in the FD patients for all the considered indexes. (a) SOAM. (b) PAD. (c) I2e.

We evaluated with the dedicated software the retinal and conjunctival vascular network of, 11 patients affected by FD, focusing first on the comparison between FD patients and controls and male vs female FD patients. The results of FD patients in comparison with the control group for the different tortuosity indexes are summarized in the following graphs (Figure 3).

In FD, retinal vessel tortuosity was higher in comparison with the control group (*p*=0.003 for SOAM, *p*=0.002 for PAD, *p* < 0.001 for I2e). On the other hand in FD conjunctival vessels were significantly more tortuous than controls only in the nasal sector (*p*=0.023 for SOAM, *p*=0.014 for PAD, *p*=0.001 for I2e) while this difference was not statistically significant in the other conjunctival sector.

We did not find differences between Fabry vs control subjects in males compared to females for all outcome measures (*p* < 0.05 for interaction term).

Linear regression analysis did not show any significant association between retinal and conjunctival vessel tortuosity indexes, retinal and conjunctival vascular parameters and increasing age (both in controls and in FD), retinal and conjunctival vessel tortuosity parameters at each sector and systemic involvement markers (in FD patients where these data were available).

## 4. Discussion

In our study, a computer-assisted analysis of retinal and conjunctival vasculature showed an increased vessel tortuosity in patients with FD. In fact the three considered tortuosity parameters (SOAM, PAD and triangular index) were significantly higher in the FD group than in controls both for retinal and nasal conjunctival vessels. These results confirm our previous report showing the value of computer-assisted evaluation of retinal vessel tortuosity in FD, but also show that the same software may be used to quantify conjunctival vessel abnormalities [28].

Among the different conjunctival sectors, the nasal sector shows a major increase of vessel tortuosity for the three considered parameters and then a significant difference in comparison with the vessels of the same sector in the control group. This result cannot be easily interpreted and may be due to some peculiar features of the vascular network in the nasal sector of the bulbar conjunctiva. We can speculate that the nasal vasculature of the conjunctiva may present a higher perfusion pressure and be more prone to arterial wall deformation determined by the bloodstream.

In our FD series vessel tortuosity was not more evident in males than in females. Even if in an X-linked disorder the phenotype is usually more severe in males than in females, our series may be too small to detect intersex differences. Moreover in FD several women may sometimes present a relevant systemic involvement [30].

The lack of a significant association between retinal and conjunctival vessel tortuosity parameters is not surprising as the two vascular regions show relevant anatomical and physiopathological differences. This result suggests that the two vascular networks should be considered independently in the evaluation of vessel tortuosity in FD. A possible option is to focus on the conjunctival nasal sector which seems more clinically interesting as it shows significant difference between FD and controls.

Retinal and conjunctival vessel tortuosity parameters did not show significant changes with increasing age. Again this may be due to the relatively small sample size in our series. Moreover, even if an increase in vessel tortuosity is expected with increasing age, this parameter has not been carefully quantified and specifically evaluated in previous longitudinal studies.

Similarly, in our series we did not observe with linear regression analysis a significant association between conjunctival and retinal vessel tortuosity parameters and the considered markers of systemic involvement in FD. Previous investigations suggested a possible predictive value of retinal vessel tortuosity on the severity of the disease [15], even if this result has not been replicated in other studies [31]. This disagreement may be due some differences in the study protocol, in the features of the study population and mainly in the procedure used to evaluate the retinal vessels course. Further studies are required to establish possible correlations between ocular vascular abnormalities and disease progression. Some recent developments in ocular vascular imaging like OCT Angiography and Adaptive Optics may offer new chances for this kind of investigation, which may provide clinically significant information for the management of FD patients. Finally, we have previously shown that vascular tortuosity parameters used in our study are reproducible, since intra- and inter-observer CV was always below 2% among normal subjects and below 2.5% among patients with FD [28].

Finally, we are aware of some limitations of our study.

It is to be noted that the sample size is relatively small, even if FD is a rare disease and patient recruitment is not easy.

Another possible bias may be represented by the difficulty to distinguish vessel abnormalities directly due to sphingolipid deposits or secondary to arterial hypertension, a vascular alteration commonly associated with FD. Primary arterial hypertension detected prior to diagnosis of FD was one of the exclusion criteria but 3 patients became hypertensive after the diagnosis of FD is made, as a complication of the disorder itself.

One also has to take into account the fact that our computer-assisted evaluation of the vasculature is not completely objective as the choice of the most tortuous vessels is dependent on the operator.

Furthermore even if in FD veins and arteries are usually affected, we did not consider them separately. We chose this option because sometimes the differentiation between the two kind of vessels is not easy. We preferred an overall evaluation of the possible vascular abnormalities, independently from the type of circulation.

The choice of the tortuosity parameters is another potential limitation. There are a number of mathematical parameters describing tortuosity [24–26]. In a previous investigation carried out by our group [28], some of the tortuosity parameters seemed more suitable than others in detecting FD retinal vascular abnormalities. We can speculate that the analysis parameters should be tailored for the specific condition or disorder under evaluation.

In conclusion, computer-assisted analysis of retinal and conjunctival vasculature showed increased vessel tortuosity in the retina and in the nasal conjunctiva, in patients with FD. This procedure may be clinically useful for the management of FD patients as it offers some quantitative scores that are potentially useful for an early diagnosis, and for monitoring the progression of the disease and its potential responses to treatment. This computer software could also be used for other vascular retinal disorders such as retinal vein occlusion or hypertensive retinopathy [32].

## Figures and Tables

**Table 1:** Principal clinical findings of our series and mutations. NYHA: New York Heart Association; MWT: interventricular maximum wall thickness; GFR: glomerular filtration rate (mL/min/1,73 mq); E.R.T: Enzyme Replacement Therapy; P.C: Pharmacological Chaperone.

	Sex	Age	NYHA	MWT (mm)	GFR (mL/min)	Stroke	Phenotype	Cornea verticillata	Mutation	Therapy
Patient 1	F	57	1	14	55.2	Yes	Classic	Yes	p.(Arg301Pro)	E.R.T.
Patient 2	F	71	1	14	76	Yes	Classic	Yes	p.(Arg301Pro)	P.C.
Patient 3	M	38	2	15	71.5	Yes	Classic	Yes	p.(Met1Val)	E.R.T.
Patient 4	F	30	1	14	110	No	Classic	No	p.(Lys240ArgfsX29)	No
Patient 5	M	65	2	21	78	No	Late-onset	No	p.(Asn215Ser)	E.R.T.
Patient 6	M	53	2	10	50	Yes	Classic	Yes	p.(Cys63Tyr)	E.R.T.
Patient 7	F	69	2	13	77	No	Classic	No	p.(Leu243Ser)	No
Patient 8	F	61	2	14	75	No	Classic	Yes	p.(Ser364Leufs^*∗*^9)	E.R.T.
Patient 9	M	35	1	12	130	Yes	Classic	Yes	p.(Ser364Leufs^*∗*^9)	E.R.T.
Patient 10	F	30	1	8	170	Yes	Classic	Yes	p.(Ser364Leufs^*∗*^9)	E.R.T.
Patient 11	M	60	1	1	19	Yes	Classic	Yes	p.(Arg112Cys)	E.R.T.

## Data Availability

Data will be made available upon request to the corresponding author.
